# Real-World Ibrexafungerp Use Patterns Among Patients with Commercial Health Insurance, United States, 2021–2023

**DOI:** 10.1007/s40801-025-00513-x

**Published:** 2025-08-16

**Authors:** Kaitlin Benedict, Dallas J. Smith, Jeremy A. W. Gold

**Affiliations:** Mycotic Diseases Branch, Division of Foodborne, Waterborne, and Environmental Diseases, National Center for Emerging and Zoonotic Infectious Diseases, Centers for Disease Control and Prevention, 1600 Clifton Road NE, Mailstop H24-9, Atlanta, GA 30329 USA

## Abstract

**Background:**

Vulvovaginal candidiasis (VVC) is a common gynecological complaint. Ibrexafungerp (brand name: Brexafemme®) is a new, first-in-class oral antifungal medication approved as a 1-day treatment for VVC and as a monthly treatment to reduce frequency of recurrent VVC.

**Objective:**

This article describes characteristics of patients who received ibrexafungerp and potential ibrexafungerp-related side effects in order to help inform optimal future use of this medication.

**Methods:**

We used a large, national commercial health insurance claims database (the Merative^TM^ MarketScan® Commercial/Medicare Database) to identify female patients with one or more outpatient ibrexafungerp prescription during 1 July 2021 to 31 December 2023. We examined patient demographic characteristics, medical conditions and medications, type of healthcare provider seen, diagnostic testing performed, and potential ibrexafungerp side effects.

**Results:**

Among 1368 female patients who received ibrexafungerp, most were also prescribed fluconazole or topical antifungals (84.7%) or had one or more vaginitis/vulvitis-related healthcare visit (76.9%) in the previous year. Few patients (2.9%) experienced a potential ibrexafungerp-related side effect.

**Conclusions:**

Our results suggest that ibrexafungerp was generally used as non-first-line treatment for VVC.

**Supplementary Information:**

The online version contains supplementary material available at 10.1007/s40801-025-00513-x.

## Key Points

**Table:** 

Ibrexafungerp is a new antifungal for treatment of vulvovaginal candidiasis (VVC), a common gynecological complaint.
In a large health insurance claims database, we identified 1368 female patients prescribed ibrexafungerp.
Most patients were previously prescribed other antifungal medication and had previous visits for VVC, suggesting that ibrexafungerp was generally used as non-first-line treatment for VVC.

## Introduction

Vulvovaginal candidiasis (VVC) is a common gynecological complaint affecting > 50% of women throughout their lifetime [1]. Diagnosing VVC can be challenging because the symptoms can resemble other common causes of vaginitis; therefore, testing is recommended to ensure proper diagnosis and treatment. Unfortunately, diagnostic testing is uncommonly performed [2, 3], resulting in potentially inappropriate empiric treatment and possibly prolonged symptoms and increased healthcare utilization [4]. VVC can typically be successfully treated using short courses of topical azoles or oral antifungal medications (primarily fluconazole), although treating severe or recurrent cases (RVVC; experienced by ~ 5% of women with VVC) or cases caused by certain non-*Candida albicans* species can be considerably more challenging [5].

Ibrexafungerp (brand name: Brexafemme®) is a first-in-class oral antifungal medication that received US Food and Drug Administration (FDA) approval in June 2021 and has been shown to be safe and effective as a 1-day treatment for VVC [6, 7]. Ibrexafungerp was also approved in December 2022 for monthly dosing to reduce RVVC frequency [8]. Clinical trials are ongoing to evaluate whether ibrexafungerp can also be used as treatment for invasive fungal infections [9]. Understanding how ibrexafungerp is currently being used in clinical practice and the frequency of side effects outside of clinical trials may help inform optimal future use of this medication. Therefore, we used a large health insurance claims database to describe characteristics of patients who received ibrexafungerp and potential ibrexafungerp-related side effects.

## Methods 

### Data Source

We used the 2021–2023 Merative^TM^ MarketScan® Commercial/Medicare Database (https://www.merative.com/content/dam/merative/documents/brief/marketscan-explainer-general.pdf). These data capture healthcare use and expenditures from inpatient stays, outpatient visits, and outpatient prescriptions, and are widely used in public health and health services research. The data are submitted by large employers and health plans and contain information for enrollees with employer-sponsored commercial health insurance and Medicare Supplemental and Medicare Advantage plans. The data represent > 30 million employees, dependents, and retirees throughout the USA.

### Study Design

We identified patients with one or more outpatient ibrexafungerp prescription during 1 July 2021 to 31 December 2023. The index date was the date of the first ibrexafungerp prescription. We selected female patients with continuous enrollment in the year before and 30 days after the index date. We examined patient demographic characteristics (e.g., age, region, urban/rural status), certain medical conditions and medications on or in the year before the index date, specialty seen and diagnostic testing performed in the 7 days surrounding the index date, and potential ibrexafungerp side effects in the 14 days after the index date. Medical conditions and potential side effects were defined using International Classification of Diseases, Tenth Revision, Clinical Modification (ICD-10-CM) codes (Online Supplementary Material Table 1). Recurrent VVC (RVVC) was defined as three or more episodes of VVC or unspecified acute vaginitis/vulvitis within 1 year, with each visit separated by ≥ 30 days.

### Statistical Analysis

We used descriptive statistics to summarize patient characteristics. Categorical variables are reported as counts and percentages, and continuous variables as medians and interquartile ranges. Analyses were conducted using Merative’s Treatment Pathways tool and SAS 9.4 (SAS Institute, Cary, NC, USA).

## Results

In total, 1938 patients (1926 female, 12 male patients) were prescribed ibrexafungerp during the study window. The final cohort comprised 1,368 female patients who met the continuous enrollment criteria. Of those, the median patient age was 38.0 years (interquartile range (IQR) 29.0–37.5); 2 (0.1%) were < 12 years old. Most patients (60.1%) were residents of the South and non-rural areas (92.7%) (Table 1). Most patients (*n* = 1072, 78.4%) received a 1-day supply of ibrexafungerp, 208 (15.2%) received a 2-day supply, and 88 (6.4%) received a ≥ 3-day supply. The median cost per 1-day supply was $464.06 (IQR $457.16–$483.44).

**Table 1:** Demographic and clinical characteristics of female patients who received ibrexafungerp

Characteristic	*n* = 1368	%
Median age, years (IQR)	38.0	(29–37.5)
Age group, years		
0–17	12	0.9%
18–34	515	37.6%
35–44	397	29.0%
45–54	280	20.5%
55–64	157	11.5%
≥ 65	7	0.5%
US Census division of primary beneficiary's residence (*n* = 1367)		
Northeast	292	21.4%
Midwest	130	9.5%
South	821	60.1%
West	124	9.1%
Rural status (*n* = 1117)		
Rural	82	7.3%
Non-rural	1,035	92.7%
Underlying conditions on or in the year before index date		
Diabetes	163	11.9%
Pregnancy^a^	17	1.2%
Immunosuppression (HIV, transplant, cancer, or medication^b^)	302	22.1%
Overweight and obesity	258	18.9%
Number of episodes of VVC or unspecified acute vaginitis/vulvitis on or in the year before index date		
0	316	23.1%
1	464	33.9%
2	297	21.7%
3 or more (i.e., RVVC)	291	21.3%
Number of visits for VVC or unspecified acute vaginitis/vulvitis on or in the year before index date		
0	316	23.1%
1	381	27.9%
2	272	19.9%
3 or more	399	29.2%
Medications in the year before index date		
Fluconazole	1,089	79.6%
Topical antifungal medication^c^	479	35.0%
Fluconazole or topical antifungal medication	1,159	84.7%
Fluconazole and topical antifungal medication	409	29.9%
Clotrimazole-betamethasone	162	11.8%
Nystatin-triamcinolone	138	10.1%
Systemic antibacterial medication	999	73.0%
Metronidazole	403	29.5%
Estrogen-containing contraceptives	348	25.4%
Hormone replacement therapy	127	9.3%
Progestin-only contraceptives	72	5.3%
Healthcare provider type visited in the 7 days before to 7 days after the index date^d^		
Obstetrician/gynecologist	546	39.9%
Family practice or internal medicine	198	14.5%
Nurse practitioner or physician assistant	169	12.4%
Acute care hospital	166	12.1%
Other type not listed above	521	38.1%
Diagnostic testing in the 7 days before to 7 days after the index date	460	33.6%
Vaginal pH test	11	0.8%
Microscopy	167	12.2%
Fungal culture	64	4.7%
Bacterial culture	76	5.6%
*Candida* nucleic acid test	264	19.3%
Antifungal susceptibility test	59	4.3%
Potential ibrexafungerp side effects in the 14 days after index date^e^	40	2.9%
Abnormal uterine and vaginal bleeding	4	0.3%
Abdominal/pelvic pain	19	1.4%
Back pain	8	0.6%
Elevation of levels of liver transaminase levels	0	0.0%
Diarrhea	3	0.2%
Dizziness	2	0.1%
Nausea/vomiting	3	0.2%
Rash	5	0.4%

*IQR* interquartile range, *VVC* vulvovaginal candidiasis, *RVVC* recurrent vulvovaginal candidiasis

^a^No women had a pregnancy diagnosis code on the index date

^b^TNF-alpha inhibitors, IL-17 and IL-23 inhibitors, mycophenolate mofetil, tacrolimus, and prednisone with ≥ 21 days’ supply

^c^Excludes shampoos and powders

^d^Patients could have visited more than one provider type on the index date

^e^These conditions were only included as side effects if the diagnosis code was not present on or in the year before index date

In total, 302 (22.1%) of patients had immunosuppression and 163 (11.9%) had diabetes on or in the year before the index date. No women had pregnancy diagnosis codes on the index date. By specialty, most patients visited an obstetrician-gynecologist (OB/GYN) (*n* = 546, 39.9%), followed by family practice or internal medicine (*n* = 198, 14.5%). VVC-related diagnostic testing was performed for 460 (33.6%) of patients, most frequently *Candida* nucleic acid testing (19.3%) and microscopy (12.2%).

Most patients (*n* = 1052, 76.9%) had ≥ 1 visit for VVC or unspecified acute vaginitis/vulvitis on or in the year before the index date; 291 (21.3%) had RVVC. Most patients (*n* = 1089, 79.6%) were prescribed fluconazole in the year before the index date; 479 (35.0%) were prescribed topical antifungal medication, 1159 (84.7%) were prescribed either fluconazole or topical antifungal medication, and 409 (29.9%) were prescribed both. Among patients who were previously prescribed fluconazole, 594 (54.5%) received three or more prescriptions. In total, 84 (6.1%) of patients prescribed ibrexafungerp were not prescribed any antifungal medication and did not have any visits for VVC or unspecified acute vaginitis/vulvitis on or in the year before the index date. In total, 286 (20.9%) were prescribed topical combination antifungal-corticosteroid medication (11.8% clotrimazole-betamethasone and 10.1% nystatin-triamcinolone).

Few (*n* = 40, 2.9%) patients prescribed ibrexafungerp experienced a potential side effect, most commonly abdominal pain (*n* = 19, 1.4%), followed by back pain (*n* = 8, 0.6%).

## Discussion

These health insurance claims-based data offer preliminary insight into how ibrexafungerp was being used among a commercially insured population during the 2.5 years after its FDA approval.

Nearly all patients who received ibrexafungerp in this analysis were adult women, the group for whom it is approved [6, 7]. In addition, over 75% of patients had a current or previous ICD-10-CM code suggesting VVC, consistent with the indication for ibrexafungerp. The high proportion of women who previously received fluconazole or a topical antifungal (85%) or had multiple VVC episodes in the previous year (77%) suggests that ibrexafungerp was often used as alternative treatment for difficult-to-treat or recurrent infections. In addition, over 20% of patients received topical antifungal-corticosteroid medication, which is concerning because these products can cause systemic side effects due to absorption of the steroid component, may be less effective compared with antifungal therapy alone, and are postulated to contribute to antifungal resistance [10–12]. In particular, clotrimazole-betamethasone, received by > 10% of patients, is contraindicated for intravaginal use [13]. Most patients received a 1-day supply of ibrexafungerp (78%); future studies with a longer follow-up period may be better suited to evaluate patients who received monthly ibrexafungerp to reduce RVVC recurrence. The high proportion of patients from the South is consistent with previously documented higher VVC rates in the South compared with other US regions [14].

Few patients (< 3%) in this analysis had diagnostic codes to suggest side effects related to ibrexafungerp. This proportion is substantially lower than the proportion of patients experiencing any side effects in clinical trials of ibrexafungerp (14.8–64.6%) [9]. These differences are likely due to study design; our analysis may underestimate the prevalence of side effects since the data represent only medically attended symptoms recorded in the medical record.

The main limitation of this analysis is that medical coding data are subject to misclassification. Additionally, the dataset did not contain information about *Candida* species or antifungal resistance testing results, which might have offered additional insight into why patients received ibrexafungerp. Data on patient race/ethnicity and socioeconomic status were also unavailable and may be useful given that ibrexafungerp is expensive (> $460 per day supply) and may eventually be used for treatment of invasive candidiasis, for which notable health disparities exist [15]. Lastly, we were unable to assess previous use of over-the-counter medications for VVC, which survey data show is common [1].

## Conclusion

Our results show that many women who received ibrexafungerp previously received multiple types and courses of fluconazole and topical combination antifungal-corticosteroid medications, emphasizing the need for improvements in diagnostic use and antifungal stewardship for VVC and RVVC.

## Supplementary Information

Below is the link to the electronic supplementary material.

Supplementary file1 (DOCX 17 KB)

## Declarations

### Availability of data

This study used third-party data that we cannot legally distribute. All relevant summary data are within the article and the supporting files. The raw data underlying the results presented are available from the MerativeTM MarketScan® Research Databases: https://www.merative.com/documents/brief/marketscan-explainer-general. Others can access the data by going to this website and contacting Merative. The authors did not have any special access privileges that others would not have.
