# Supplementary material for: Real-World Ibrexafungerp Use Patterns Among Patients with Commercial Health Insurance, United States, 2021–2023
Source: Drugs Real World Outcomes. 2025 Aug 16;12(4):563–7. doi: 10.1007/s40801-025-00513-x (PMC12634974; doi:10.1007/s40801-025-00513-x)
Supplement: Supplementary file 1 — Supplementary file1 (DOCX 17 KB) [file 40801_2025_513_MOESM1_ESM.docx]

**Real-world ibrexafungerp use patterns among patients with commercial health insurance, United States, 2021–2023**

**Authors:** Kaitlin Benedict, Dallas J. Smith, Jeremy A.W. Gold

**Author affiliation:** Mycotic Diseases Branch, Division of Foodborne, Waterborne, and Environmental Diseases, National Center for Emerging and Zoonotic Infectious Diseases, Centers for Disease Control and Prevention, Atlanta, Georgia, USA

**Supplementary Table.** International Classification of Diseases, Tenth Revision, Clinical Modification (ICD-10-CM) and Current Procedural Terminology (CPT) codes used to identify conditions and features of interest

| **Description** | **ICD-10-CM code(s)** |
| --- | --- |
| Abdominal/pelvic pain | R10 |
| Acute vaginitis or vulvitis | N76.0, N76.2, N76.81, N76.89 |
| Back pain | M54 |
| Cancer | C00–C96, excluding C44 |
| Diabetes | E08–E13 |
| Diarrhea | R19.7 |
| Dizziness | R42 |
| Elevation of levels of liver transaminase levels | R74.01 |
| HIV/AIDS | B20, Z21 |
| Nausea/vomiting | R11 |
| Overweight and obesity | E66 |
| Pregnancy | Z33.1, Z33.3, Z34, O09 |
| Rash | R21 |
| Solid organ or stem cell transplant or transplant complications | T86, Z94 (excluding Z94.7), Z95.2, Z95.3 |
| Unspecified abnormal uterine and vaginal bleeding | N93.8, N93.9 |
| Vulvovaginal candidiasis | B37.3 |
| **Description** | **CPT code(s)** |
| Point of care testing |  |
| KOH test or smear | 87220, 87205, 87206, 87210 |
| Vaginal pH test | 82120 |
| Laboratory-based testing |  |
| Fungal culture | 87101, 87102, 87106, 87107 |
| Bacterial culture | 87070, 87071, 87073, 87075, 87076, 87077 |
| *Candida* nucleic acid test | 87480, 87481, 87482 |
| Antifungal susceptibility testing | 87186 |
